# Supplementary material for: Electron-phonon coupling and vibrational properties of size-selected linear carbon chains by resonance Raman scattering
Source: Nat Commun. 2022 Aug 27;13:5052. doi: 10.1038/s41467-022-32801-3 (PMC9420137; doi:10.1038/s41467-022-32801-3)
Supplement: Supplementary file 1 — Supplementary Information [file 41467_2022_32801_MOESM1_ESM.pdf]

Supplementary Information: Electron-phonon coupling and vibrational properties of size-selected linear carbon chains by resonance Raman scattering

P. Marabotti[1], M. Tommasini[2], C. Castiglioni[2], P. Serafini[1], S. Peggiani[1], M. Tortora[3], B. Rossi[3], A. Li Bassi[1], V. Russo[1], and C. S. Casari[1]

[1]Micro and Nanostructured Materials Laboratory - NanoLab, Department of Energy, Politecnico di Milano via Ponzio 34/3, I-20133, Milano, Italy
[2]Department of Chemistry, Materials and Chem. Eng. 'G. Natta', Politecnico di Milano Piazza Leonardo da Vinci 32, I-20133, Milano, Italy
[3]Elettra Sincrotrone Trieste, S.S. 114 km 163.5, Basovizza, 34149 Trieste, Italy

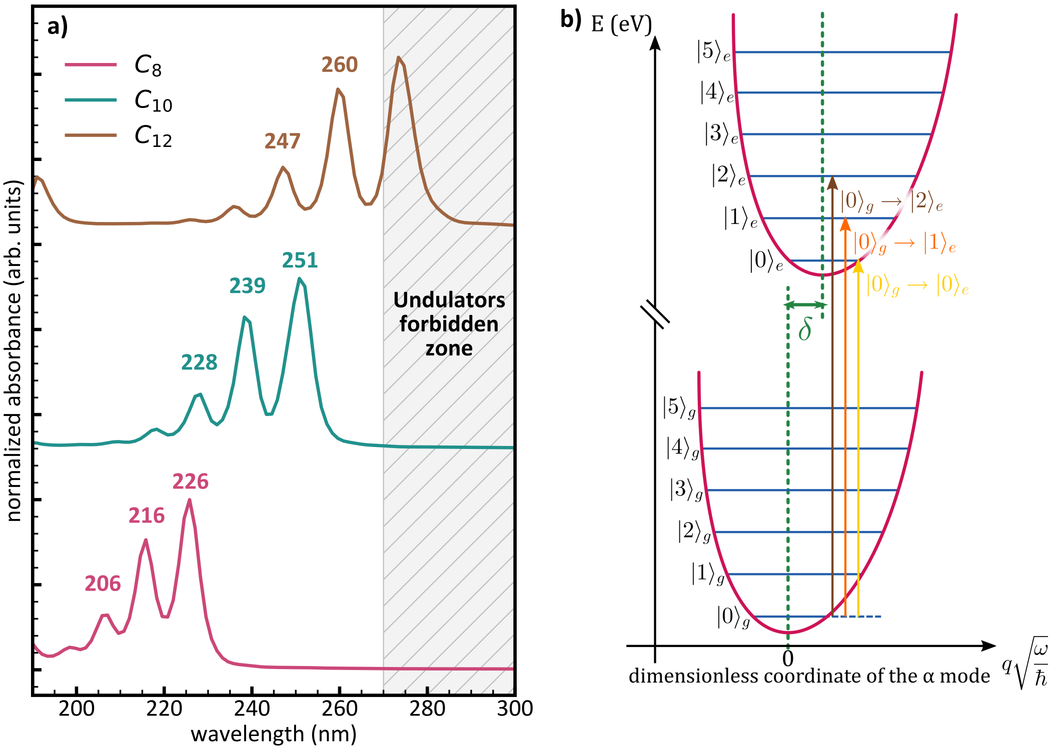

Supplementary Figure 1. **UV-Vis absorption spectra of C_8, C_{10}, and C_{12}, and schematic diagram of vibronic transitions.** **a**) UV absorption spectra of size-selected H-capped polyyne with 8, 10, and 12 carbon atoms (namely C_8, C_{10}, and C_{12}), extracted from the spectrophotometer integrated into the HPLC apparatus. The greyed region above 270 nm indicates the excitation energies not accessible with the characteristics of the undulator of the beamline. **b**) Schematic diagram of the vibrational levels in ground ($|i\rangle_g$) and excited ($|k\rangle_e$) electronic states. Yellow, orange and brown lines indicate the first three electronic transitions corresponding to the first three peaks of the vibronic pattern in the absorption spectra of panel a). The displacement parameter δ is the distance between the minima of the potential energy surfaces.

Molecule	Concentration [mol/L]	Absorption peaks [nm]	Power on the sample [μW]
C_8	$\approx 6.21 \cdot 10^{-6}$	226 nm (k=0)	22.0
		216 nm (k=1)	16.2
		206 nm (k=2)	4.5
C_{10}	$\approx 2.32 \cdot 10^{-6}$	251 nm (k=0)	17.6
		239 nm (k=1)	22.1
		226 nm (k=2)	22.0
C_{12}	$\approx 1.48 \cdot 10^{-6}$	273 nm (k=0)	–
		261 nm (k=1)	18.0
		247 nm (k=2)	21.6

Supplementary Table 1. **Concentrations and absorption peaks of polyynes and power of the Raman excitation beam.** Concentrations in mol/L of C_8, C_{10}, and C_{12} in water-acetonitrile solutions and excitation wavelengths corresponding to their $|0\rangle_g \rightarrow |k\rangle_e$ vibronic transitions. The integer k is the vibrational quantum number of the excited vibrational label involved (see text). The power deposited on the sample at each wavelength is also reported. The Raman measurement corresponding to the excitation at the 1st maximum of C_{12} ($|0\rangle_g \rightarrow |0\rangle_e$) could not be performed due to limitations in the undulator gap aperture.

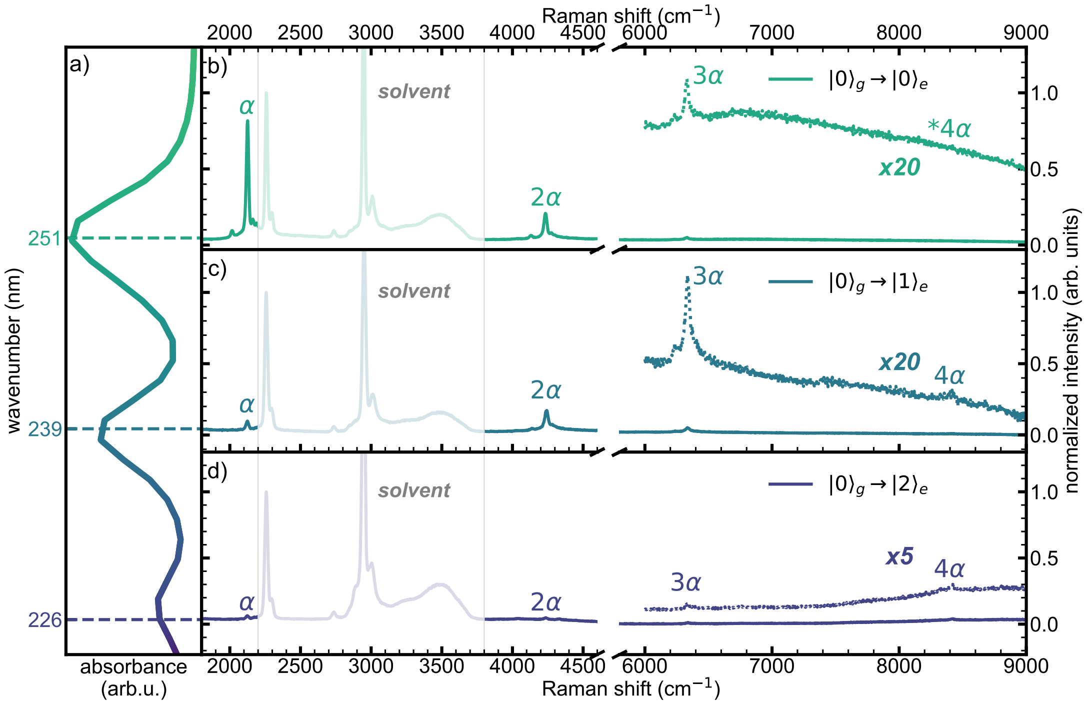

Supplementary Figure 2. **UV-Vis absorption spectrum and UV resonance Raman spectra of C_{10}.** UV-Vis absorption spectrum (panel **a**) and UV resonance Raman spectra of C_{10}, excited with synchrotron radiation at three different wavelengths: 251 nm ($|0\rangle_g \rightarrow |0\rangle_e$, panel **b**), 239 nm ($|0\rangle_g \rightarrow |1\rangle_e$, panel **c**), and 226 nm ($|0\rangle_g \rightarrow |2\rangle_e$, panel **d**). The signals above 6000 cm^{-1} in all panels are magnified to highlight low-intensity signals. The vibrational lines of the solvent are shadowed by white boxes. The spectra are normalized to the CN stretching peak of acetonitrile.

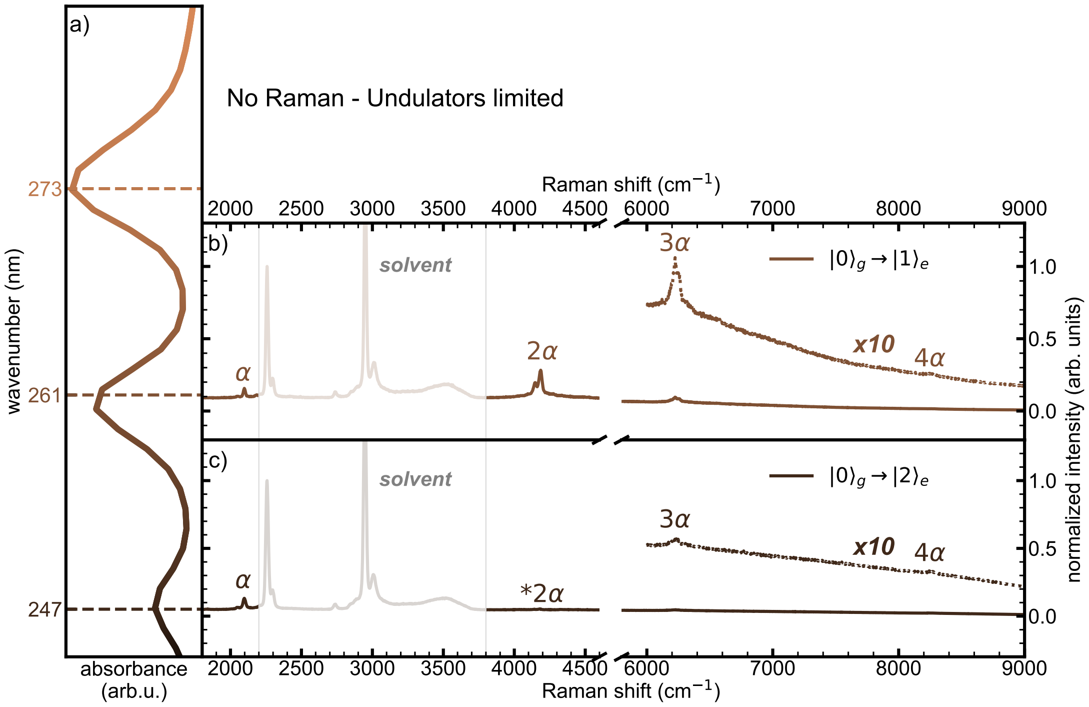

Supplementary Figure 3. **UV-Vis absorption spectrum and UV resonance Raman spectra of C_{12}.** UV-Vis absorption spectrum (panel **a**) and UV resonance Raman spectra of C_{12}, excited with synchrotron radiation at three different wavelengths: 261 nm ($|0\rangle_g \rightarrow |1\rangle_e$, panel **b**) and 247 nm ($|0\rangle_g \rightarrow |2\rangle_e$, panel **c**). The excitation corresponding to the absorption maximum of C_{12} (273 nm, $|0\rangle_g \rightarrow |0\rangle_e$) could not be performed due to limitations in the undulator gap aperture. The signals above 6000 cm^{-1} in all panels are magnified to highlight low-intensity signals. The vibrational lines of the solvent are shadowed by white boxes. The spectra are normalized to the CN stretching peak of acetonitrile.

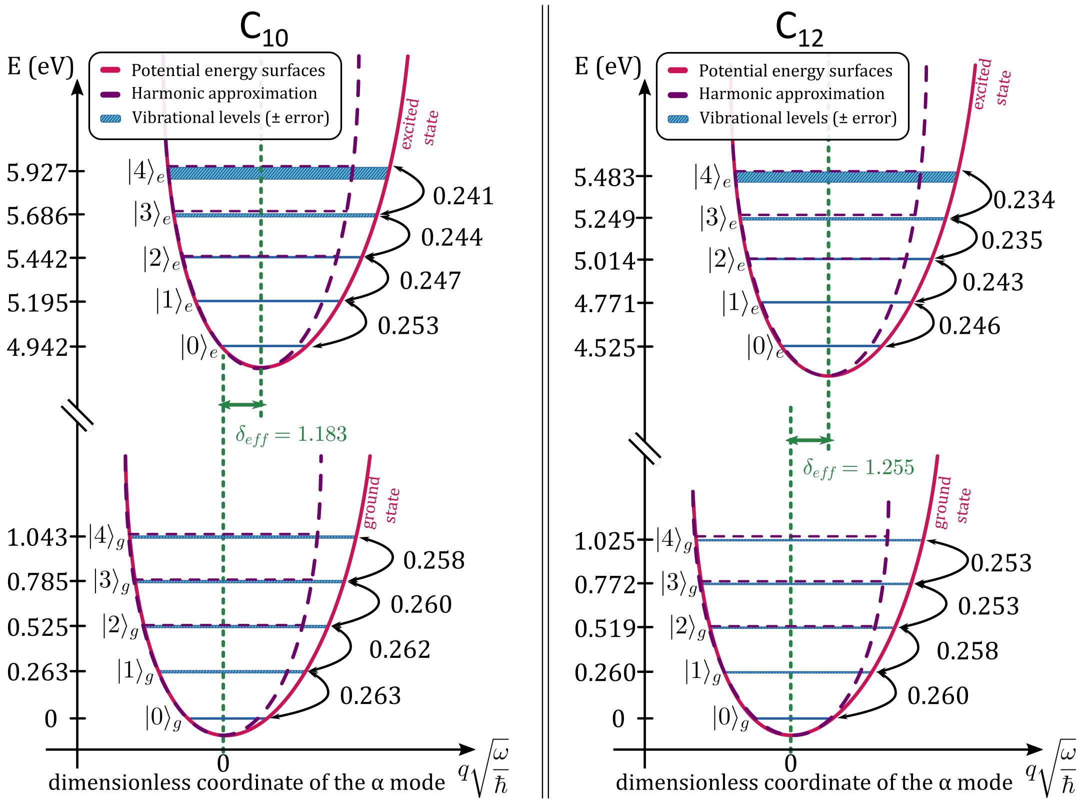

Supplementary Figure 4. **Vibrational energy diagrams of ground and excited states of the α mode of C_{10} and C_{12}.** Vibrational diagram of the ground and excited potential energy surfaces extracted from experimental UVRR and UV-Vis absorption spectra and referred to the normal coordinate q of the α mode of C_{10} and C_{12}. The energy (in eV) of each vibrational level $|k\rangle_j$ of the jth state is reported in the left axis, while the energy of the $|0\rangle_g$ level is fixed to 0 eV. The thickness of the vibronic lines represents the error of the fit model used to deconvolve the UVRR and UV-Vis absorption spectra. The spacing between neighboring vibrational levels ($\Omega_{k,j}$) is displayed on the right in eV and indicated by black arrows. The purples dashed curves and lines represent the harmonic approximation of the potential energy surfaces and vibrational levels, respectively. The effective displacement parameter δ_{eff} is reported in green.

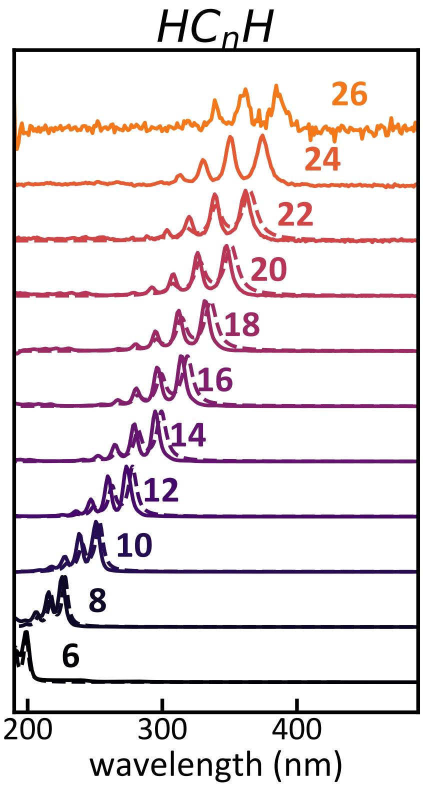

Supplementary Figure 5. **UV-Vis absorption spectra of H-capped polyynes.** UV-Vis absorption spectra of H-capped polyynes (HC_nH) ranging from 6 to 26 carbon atoms. The dashed lines are calculated UV-Vis spectra.

Supplementary Discussion

Resonance Raman intensities of polyynes: analytic expression and prediction

In this Section of the Supplementary Information we analyze in details the resonance Raman and UV-Vis absorption spectra of the three hydrogen capped polyynes of interest (HC_nH or C_n, $n = 8,10,12$). The final aim of this Section is the prediction of the intensities of the overtones of the α mode obtained through UV resonance Raman spectroscopy. Achieving this goal implies determining the displacement parameters (δ) that are required for the evaluation of the Franck-Condon factors appearing in the expression of the resonance Raman polarizability tensor introduced by Albrecht [1]. Remarkably, such parameters determine both the relative intensities of the vibronic progressions observed in the UV-Vis absorption spectra of polyynes [2] and the relative Raman intensities of

the two CC stretching modes (denoted α and β – [2, 3]).

For a given k-th normal coordinate of interest, associated to a vibration with angular frequency ω_k, the dimensionless displacement parameter δ_k is the difference between the equilibrium position in the ground state and in the excited state [4], projected along that normal coordinate (further details can be found in [5]):

$$\delta_k = \left(\frac{\omega_k}{\hbar}\right)^{1/2} [\boldsymbol{X}^g - \boldsymbol{X}^e]^t \boldsymbol{M}^{1/2} \boldsymbol{L}_k \tag{1}$$

In Equation (1) the t superscript denotes transposition, $\boldsymbol{X}^e$ ($\boldsymbol{X}^g$) is the column vector containing the ordered sequence of the $3N$ Cartesian coordinates of the N atoms of the molecule in the equilibrium structure of the electronically excited (ground) state, $\boldsymbol{M}$ is the $3N \times 3N$ diagonal matrix of the atomic masses associated to each Cartesian coordinate, and $\boldsymbol{L}_k$ is the k-th column eigenvector of the secular equation describing molecular vibrational dynamics within the harmonic approximation [6]:

$$\left[\boldsymbol{M}^{-1/2}\boldsymbol{K}\boldsymbol{M}^{-1/2}\right] \boldsymbol{L}_k = \omega_k^2 \boldsymbol{L}_k \tag{2}$$

In Equation (2) $\boldsymbol{K}$ is the Hessian matrix of the potential energy of the molecule (in the ground state) with respect to the displacements of the nuclear coordinates, namely $K_{ij} = \partial^2 V/\partial X_i \partial X_j$. We finally mention that the δ displacement parameters are used in [4, 5] to compute the Franck-Condon factors that are central in the formulation of resonance Raman theory [1].
It can be shown that the Huang-Rhys factor S_k of a given mode is related to the displacement parameter δ_k by the following simple relation [5]:

$$S_k = \frac{\delta_k^2}{2} \tag{3}$$

This result is easily shown by considering the expression of the Franck-Condon integral in different notations, namely: $|\langle 0|0\rangle|^2 = e^{-S}$ [7]$= e^{-\delta_k^2/2}$ [4, 5].

The resonance Raman scattering tensor

In resonance conditions with a given molecular excited state e, according to Albrecht, the Raman scattering tensor is led by the so-called A-term [1]:

$$A \equiv \boldsymbol{\alpha}(m = gi \rightarrow n = gj) = \boldsymbol{\mu}_{ge} \otimes \boldsymbol{\mu}_{eg} \sum_v \left[{}_g\langle i|v\rangle_e \, F_v \, {}_e\langle v|j\rangle_g\right] \tag{4}$$

where $\boldsymbol{\mu}_{ge}$ and $\boldsymbol{\mu}_{eg}$ are the electronic transition dipole moment from the ground to the excited state and vice versa, respectively; $|i\rangle_g$ represents the initial vibrational level in the electronic ground state, $|v\rangle_e$ is the vibrational level of the electronic excited state reached during the resonance process and $|j\rangle_g$ is the final vibrational level in the electronic ground state. The resonance term F_v is expressed as

$$F_v = \frac{1}{[E_e^0 + \hbar\Omega_e(v + 1/2)] - \left[E_g^0 + \hbar\Omega_g(i + 1/2)\right] - \hbar\omega_0 + \mathrm{i}\gamma} \tag{5}$$

where E_g^0 and E_e^0 are the energy minima of the ground and excited states, respectively; $\hbar\Omega_g$ and $\hbar\Omega_e$ are the energy spacing of the harmonic vibrational levels of the ground and excited states, respectively; $\hbar\omega_0$ is the energy of the exciting radiation and γ is the damping factor for the electronic state e. By considering a direct proportionality between the energy spacing of the vibrational levels of the excited and ground states as $\Omega_e = \alpha^2\Omega_g$ ($\Omega_g = \alpha^{-2}\Omega_e$), where α^2 is the proportionality constant (for details see [4]), one gets:

$$F_v = \frac{1}{\left[E_e^0 + \hbar\Omega_e(v+1/2)\right] - \left[E_g^0 + \alpha^{-2}\hbar\Omega_e(i+1/2)\right] - \hbar\omega_0 + \mathrm{i}\gamma} \tag{6}$$

so that:

$$\begin{aligned} F_v &= \frac{1}{\left[\Delta E_{ge}^0 - \hbar\omega_0\right] + \hbar\Omega_e\left[(v+1/2) - \alpha^{-2}(i+1/2)\right] + \mathrm{i}\gamma} = \\ &= \frac{1}{\left[\Delta E_{ge}^0 - \hbar\omega_0\right] + \hbar\Omega_e\left[(v-\alpha^{-2}i) + \frac{1}{2}(1-\alpha^{-2})\right] + \mathrm{i}\gamma} \end{aligned} \tag{7}$$

where $\Delta E_{ge}^0 = E_e^0 - E_g^0$.
As initial state, we can assume $i = 0$ since the α modes of polyynes ($\approx 1800-2200\ cm^{-1}$) are at much higher energies compared to the ambient thermal energy. The resonance condition with the vibronic progression $|0\rangle_g \rightarrow |0\rangle_e$ (P), $|0\rangle_g \rightarrow |1\rangle_e$ (I), $|0\rangle_g \rightarrow |2\rangle_e$ (II), ..., $|0\rangle_g \rightarrow |k\rangle_e$ (k) in the absorption spectrum implies photon energies $\hbar\omega_0$ that satisfy:

$$\begin{aligned} \hbar\omega_0 &= \left[E_e^0 + \hbar\Omega_e(k+1/2)\right] - \left[E_g^0 + \alpha^{-2}\hbar\Omega_e\frac{1}{2}\right] = \\ &= \Delta E_{ge}^0 + \hbar\Omega_e\left[k + \frac{1}{2}\left(1-\alpha^{-2}\right)\right] \end{aligned} \tag{8}$$

By substituting $\hbar\omega_0$ given by Equation (9) into Equation (8), we obtain the expression of F_v in resonance with the $|0\rangle_g \rightarrow |k\rangle_e$:

$$F_v = \frac{1}{\hbar\Omega_e\left[v-k\right] + \mathrm{i}\gamma} \tag{9}$$

Thus, the leading F_v term corresponds to $v = k$, which allows to introduce the following compact expression of the vibronic transition Raman tensor in resonance with the $|0\rangle_g \rightarrow |k\rangle_e$:

$$\boldsymbol{\alpha}(0 \rightarrow m)_{0k} \approx (\mathrm{i}\gamma)^{-1}\boldsymbol{\mu}_{ge} \otimes \boldsymbol{\mu}_{eg}\left[{}_g\langle 0|k\rangle_e\, {}_e\langle k|m\rangle_g\right] \tag{10}$$

where $|m\rangle_g$ is the final vibrational level in the electronic ground state.

Analysis of the resonance Raman spectra of C_8, C_{10}, and C_{12}

The first-order resonance Raman spectra of H-capped polyynes is characterized by definite α and β lines [2, 3], also when excited in resonance conditions thanks to the Elettra

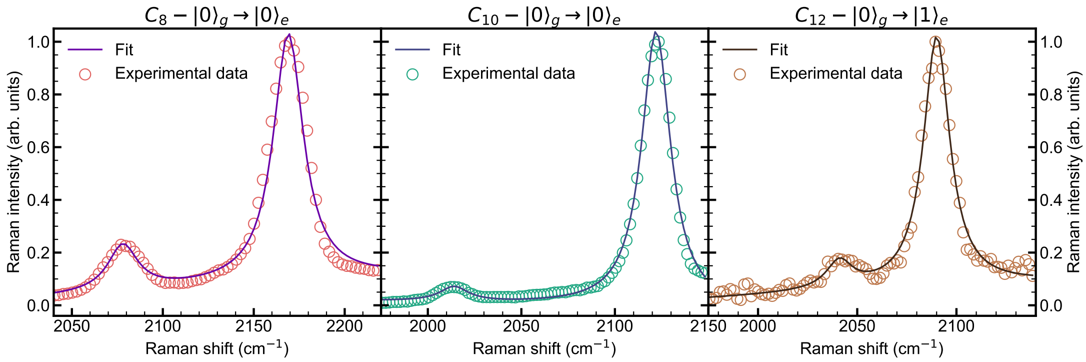

Supplementary Figure 6. **First-order UV resonance Raman spectra of polyynes.** Experimental first-order resonance Raman spectra of C_8 (226 nm – 5.49 eV), C_{10} (251 nm – 4.94 eV), C_{12} (261 nm – 4.75 eV) measured in a mixture of water and acetonitrile (dotted circles). The best fit of each spectrum is reported as a solid line in the corresponding panel.

setup (Figure 6). The spectra can be fitted reasonably well by a model comprising two Lorentzian lineshapes and a linear baseline. The results of the fitting procedure are reported in Table 2. From such fitting one can straightforwardly determine the experimental values of the relative Raman intensities of the α and β lines that will be required below. It is important to notice that C_8 and C_{10} are excited at their corresponding $|0\rangle_g \rightarrow |0\rangle_e$ transitions, whereas C_{12} is probed at its $|0\rangle_g \rightarrow |1\rangle_e$ transition due to the limitation to the synchrotron radiation imposed by the undulators.

Molecule	$\bar{\nu}_1$ (α, cm^{-1})	$\bar{\nu}_2$ (β, cm^{-1})	$\phi = I_2/I_1$	FWHM (cm^{-1})
C_8	2169	2078	0.19	20
C_{10}	2122	2014	0.05	19
C_{12}	2090	2041	0.11	16

Supplementary Table 2. **Parameters obtained from the best fit of the first order resonance Raman spectra of the investigated polyynes.** Positions of the α and β modes, intensity ratios between β and α modes, and full width half maximum as extracted from the best fit of the first order resonance Raman spectra of C_8, C_{10}, and C_{12} of Figure 6

Analysis of the UV-Visible absorption spectra of C_8, C_{10}, and C_{12}

The absorption spectra of H-capped polyynes show definite vibronic progressions [2] that can be fitted, to a good approximation, with a multi-Lorentzian model that includes a

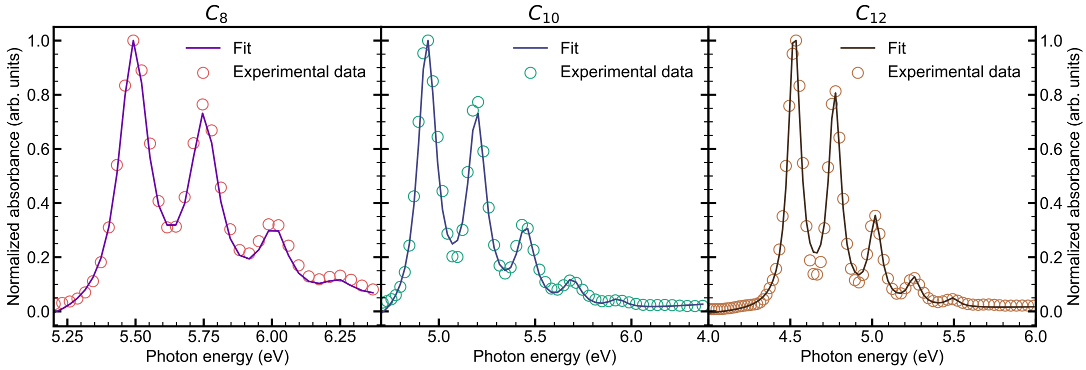

Supplementary Figure 7. **UV-Vis absorption spectra of polyynes.** UV-Vis absorption spectra of C_8, C_{10}, C_{12} measured in a mixture of water and acetonitrile (dotted point). The best fit of each polyyne is reported as a solid line. The optical path is 1 cm.

linear baseline correction. The results for C_8, C_{10}, and C_{12} are reported in Figure 7.

Molecule	P (eV)	I (eV)	II (eV)	III (eV)	IV (eV)	I_I/I_P (ξ)	I_{II}/I_P	I_{III}/I_P	I_{IV}/I_P	FWHM (eV)
C_8	5.493	5.751	6.010	6.243	–	0.68	0.25	0.06	–	0.13
C_{10}	4.939	5.197	5.451	5.693	5.927	0.70	0.29	0.10	0.03	0.12
C_{12}	4.525	4.772	5.020	5.256	5.493	0.75	0.31	0.10	0.03	0.09

Supplementary Table 3. **Parameters obtained from the best fit of the UV-Vis absorption spectra of polyynes.** Energies of the vibronic maxima, ratio of absorbances, and full width at half maximum of C_8, C_{10}, and C_{12} of Figure 7.

To obtain information about the values of the nondimensional displacement parameters (δ) it is useful to carry out, within the Franck-Condon approximation, the analysis of the $|0\rangle_g \rightarrow |0\rangle_e$ (P) and $|0\rangle_g \rightarrow |1\rangle_e$ (I) bands. We limit ourselves to *two* Raman active modes with appreciable δ. We label 1 and 2 such modes that correspond, respectively, to the α and β lines of hydrogen capped polyynes, so that δ_1 and δ_2 are the nondimensional displacement parameters for the α and β modes, respectively [2, 3]. For a given k-th resonance ($k = 0, 1, 2, ...$) in the UV-Vis absorption spectrum the transition energy can be written as:

$$\Delta E^{1,2} = \left(E_e^0 - E_g^0\right) + \hbar\Omega_e^{1,2}\,(k + 1/2) - \hbar\Omega_g^{1,2}\,(0 + 1/2) \qquad (11)$$

where we have assumed to start from the $i = 0$ vibrational level of the ground state and the apices 1 and 2 indicate the α and β modes as declared before. Since $\hbar\Omega_e^{1,2} = \alpha_{1,2}^2\hbar\Omega_g^{1,2}$

(see Section 2), we have:

$$\Delta E^{1,2} = \Delta E^0_{ge} + \hbar\Omega_g^{1,2}\left[\frac{1}{2}\left(\alpha^2_{1,2} - 1\right) + \alpha^2_{1,2}k\right] \tag{12}$$

where ΔE^0_{ge} is the difference between the energy minima of the electronic ground and excited states. Given that:

(i) $\alpha_{1,2}$ is close to one, *i.e.*, the vibrational frequency in the excited state is close to that in the ground state;

(ii) ΔE^0_{ge} is significantly larger than $\hbar\Omega_g^{1,2}$, *i.e.*, electronic transitions lay in the UV range whereas vibrational transitions are in the mid-IR range;

(iii) the difference between Ω^1_g and Ω^2_g (observed in resonance Raman) is comparable with the apparent linewidth of the vibronic transitions (Figure 7),

by considering Equation (12) one fully justifies the experimental observation of vibronic progressions that are *not* resolved as individual transitions involving the α and β normal modes of polyynes. Therefore, the *total* intensity of a given vibronic manifold is associated *as a whole* to the square of the multidimensional Franck-Condon factor (${}_g\langle\mathbf{0}|\mathbf{0}\rangle_e$, ${}_g\langle\mathbf{0}|\mathbf{1}\rangle_e$, ${}_g\langle\mathbf{0}|\mathbf{2}\rangle_e$, ...; where $\mathbf{1}, \mathbf{2}, ...$ denote vibrationally excited state containing one, two, ... vibrational quanta distributed over the relevant normal modes – here just two). Hence, the intensities of the $|0\rangle_g \to |0\rangle_e$ (P) and $|0\rangle_g \to |1\rangle_e$ (I) transitions are given by the following expressions:

$$I_P \propto |{}_g\langle\mathbf{0}|\mathbf{0}\rangle_e|^2 = |{}_g\langle 0_1|0_1\rangle_e|^2 \;\; |{}_g\langle 0_2|0_2\rangle_e|^2 \tag{13}$$
$$I_I \propto \sum_{\mathbf{1}} |{}_g\langle\mathbf{0}|\mathbf{1}\rangle_e|^2 = |{}_g\langle 0_1|1_1\rangle_e|^2 \;\; |{}_g\langle 0_2|0_2\rangle_e|^2 + |{}_g\langle 0_1|0_1\rangle_e|^2 \;\; |{}_g\langle 0_2|1_2\rangle_e|^2$$

where the symbol $\sum_{\mathbf{1}}$ implies a summation over all the possible one-quantum states, in our case these are $|1_1\rangle|0_2\rangle$ and $|0_1\rangle|1_2\rangle$. We then introduce the experimentally available intensity ratio ξ:

$$\xi = I_I/I_P = \frac{|{}_g\langle 0_1|1_1\rangle_e|^2 \;\; |{}_g\langle 0_2|0_2\rangle_e|^2 + |{}_g\langle 0_1|0_1\rangle_e|^2 \;\; |{}_g\langle 0_2|1_2\rangle_e|^2}{|{}_g\langle 0_1|0_1\rangle_e|^2 \;\; |{}_g\langle 0_2|0_2\rangle_e|^2} = \tag{14}$$
$$= \left|\frac{{}_g\langle 0_1|1_1\rangle_e}{{}_g\langle 0_1|0_1\rangle_e}\right|^2 + \left|\frac{{}_g\langle 0_2|1_2\rangle_e}{{}_g\langle 0_2|0_2\rangle_e}\right|^2$$

By recalling that $\langle 0|0\rangle = \exp(-\delta^2/4)$ and $\langle 0|1\rangle = \exp(-\delta^2/4)\,\delta/\sqrt{2}$ [4] and labelling the nondimensional parameters of the α and β modes as δ_1 and δ_2, respectively, one obtains:

$$\xi = I_I/I_P = \frac{\delta_1^2}{2} + \frac{\delta_2^2}{2} = \frac{\delta^2_{eff}}{2} \tag{15}$$

The last equality establishes the correspondence between the two-modes description of the vibronic progression and the effective one-mode representations (see main text).

Determination of δ_1 and δ_2 from the joint analysis of Raman and UV-Vis data

$|\mathbf{0}\rangle_\mathbf{g} \rightarrow |\mathbf{0}\rangle_\mathbf{e}$ (P) resonance

The ratio of the Raman intensities of the fundamental transitions (α, 1; β, 2) are another useful source of information to fix the values of the δ parameters, in addition to Equation (15) above. Based on Equation (10), for a fundamental transition ($m = 1$), and in resonance with the $|0\rangle_g \rightarrow |0\rangle_e$ (P) transition (*i.e.*, $k = 0$), the Raman scattering tensor is written as:

$$\boldsymbol{\alpha}(0 \rightarrow 1)_{00} \approx (\mathrm{i}\gamma)^{-1} \boldsymbol{\mu}_{ge} \otimes \boldsymbol{\mu}_{eg} \left[{}_g\langle 0|0\rangle_e \, {}_e\langle 0|1\rangle_g \right] \tag{16}$$

Therefore the Raman intensity ratio ϕ between the intensity of the β (2) and α (1) lines is given by:

$$\phi = I_2/I_1 = \left| \frac{{}_g\langle 0_2|0_2\rangle_e \, {}_e\langle 0_2|1_2\rangle_g}{{}_g\langle 0_1|0_1\rangle_e \, {}_e\langle 0_1|1_1\rangle_g} \right|^2 = \frac{\delta_2^2}{\delta_1^2} \frac{\exp(-\delta_2^2)}{\exp(-\delta_1^2)} \tag{17}$$

Given the intensity ratios obtained from UV-Vis absorption (ξ) and resonance Raman (ϕ) spectra, one has to cope with the following set of equations:

$$\xi = \frac{\delta_1^2}{2} + \frac{\delta_2^2}{2} \tag{18}$$

$$\phi = \frac{\delta_2^2}{\delta_1^2} \frac{\exp(-\delta_2^2)}{\exp(-\delta_1^2)} = \frac{\delta_2^2}{\delta_1^2} \exp(\delta_1^2 - \delta_2^2) \tag{19}$$

The first equation gives $\delta_2^2 = 2\xi - \delta_1^2$. By substituting this expression of δ_2^2 in the second equation, one obtains an equation that can be numerically solved for δ_1 based on the known values of ϕ and ξ (see Section below):

$$\phi + \frac{\delta_1^2 - 2\xi}{\delta_1^2} \exp(2\delta_1^2 - 2\xi) = 0 \tag{20}$$

$|\mathbf{0}\rangle_\mathbf{g} \rightarrow |\mathbf{1}\rangle_\mathbf{e}$ (I) resonance

Based on Equation (10), for a fundamental transition ($m = 1$) and in resonance with the $|0\rangle_g \rightarrow |1\rangle_e$ (I) vibronic transition (*i.e.*, $k = 1$) the Raman scattering tensor is written as:

$$\boldsymbol{\alpha}(0 \rightarrow 1)_{01} \approx (\mathrm{i}\gamma)^{-1} \boldsymbol{\mu}_{ge} \otimes \boldsymbol{\mu}_{eg} \left[{}_g\langle 0|1\rangle_e \, {}_e\langle 1|1\rangle_g \right] \tag{21}$$

By recalling that $\langle 0|1\rangle = \exp(-\delta^2/4)\delta/\sqrt{2}$ and $\langle 1|1\rangle = \exp(-\delta^2/4)(2-\delta^2)/2$ the Raman intensity ratio ϕ is given by:

$$\phi = I_2/I_1 = \left| \frac{{}_g\langle 0_2|1_2\rangle_e \, {}_e\langle 1_2|1_2\rangle_g}{{}_g\langle 0_1|1_1\rangle_e \, {}_e\langle 1_1|1_1\rangle_g} \right|^2 = \frac{\exp(-\delta_2^2)\, \delta_2^2 (2 - \delta_2^2)^2}{\exp(-\delta_1^2)\, \delta_1^2 (2 - \delta_1^2)^2} \tag{22}$$

The equations describing the intensity ratios obtained from UV-Vis absorption (ξ) and resonance Raman (ϕ) spectra are the following:

$$\xi = \frac{\delta_1^2}{2} + \frac{\delta_2^2}{2} \tag{23}$$

$$\phi = \frac{\delta_2^2 (2 - \delta_2^2)^2}{\delta_1^2 (2 - \delta_1^2)^2} \exp(\delta_1^2 - \delta_2^2) \tag{24}$$

The first equation gives $\delta_2^2 = 2\xi - \delta_1^2$. By substituting this expression of δ_2^2 in the second equation, one obtains:

$$\phi + \frac{(\delta_1^2 - 2\xi)(\delta_1^2 - 2\xi + 2)^2}{\delta_1^2(\delta_1^2 - 2)^2} \exp(2\delta_1^2 - 2\xi) = 0 \tag{25}$$

Equation (25) can be numerically solved for δ_1 based on the known values of ϕ and ξ (see Section).

Assessment of the numerical values of δ_1 and δ_2

Given the values of the ξ and ϕ parameters determined from UV-Vis and Raman intensity ratios, the numerical solution of Equation (20) (for P-resonance Raman data) or Equation (25) (for I-resonance Raman data) can be straightforwardly carried out (*e.g.*, by the fsolve function implemented in Matlab [8]). This allows the determination of the numerical value of δ_1. Thereafter, δ_2 is given by $\delta_2 = \sqrt{2\xi - \delta_1^2}$. The numerical results of such procedure are collected in Table 4, and have been obtained by applying the formulas valid for P- or I-resonance, as indicated in Table 4.

Molecule	δ_1	δ_2	resonance
C_8	1.136	0.266	$\lvert 0\rangle_g \rightarrow \lvert 0\rangle_e$ (P)
C_{10}	1.178	0.137	$\lvert 0\rangle_g \rightarrow \lvert 0\rangle_e$ (P)
C_{12}	1.228	0.047	$\lvert 0\rangle_g \rightarrow \lvert 1\rangle_e$ (I)

Supplementary Table 4. **Effective displacement parameters δ_1 and δ_2 of polyynes.** Values of δ_1 obtained from the numerical solution of Equation (25) – for I-resonance, or Equation (20) – for P-resonance. The values of the ξ and ϕ parameters (required for the numerical solution) are reported in Table 3 and Table 2, respectively. Independently from the resonance condition, δ_2 is given by $\sqrt{2\xi - \delta_1^2}$ (see Equation (18)).

Analysis of the progression of the overtones of the α line

As reported in Figure 2 in the main text, namely the Raman spectrum of C_8 measured in resonance with the $|0\rangle_g \rightarrow |0\rangle_e$ (P) transition, several overtones and combinations of the α and β lines can be identified in the experimental resonance Raman spectrum. It is therefore interesting to rationalize the observed decreasing behavior of the Raman intensities based on Albrecht theory. Based on Equation (10), and for a given choice of the excitation resonance condition ($|0\rangle_g \rightarrow |k\rangle_e$), one can introduce the ratio of the Raman intensity of a given overtone ($m > 1$) with respect to the fundamental transition ($m = 1$) – we take into account the well-known dependence of Raman intensity upon the fourth power of the frequency of the scattered photon (for the Stokes process):

$$R_{km} = \frac{I(0 \rightarrow m)_{0k}}{I(0 \rightarrow 1)_{0k}} = \left[\frac{\omega_0 - m\Omega_\alpha}{\omega_0 - \Omega_\alpha}\right]^4 \left[\frac{{}_g\langle 0|k\rangle_e \, {}_e\langle k|m\rangle_g}{{}_g\langle 0|k\rangle_e \, {}_e\langle k|1\rangle_g}\right]^2 = \left[\frac{1 - m\epsilon}{1 - \epsilon}\right]^4 \left[\frac{{}_e\langle k|m\rangle_g}{{}_e\langle k|1\rangle_g}\right]^2 \tag{26}$$

where $\epsilon \equiv \Omega_\alpha/\omega_0$ is a small number ($\approx 5 \cdot 10^{-2}$) for all the UV excitations here considered (ω_0) and the typical values of the position of the α-line (Ω_α). The formula given by Equation (26) can be conveniently evaluated by adopting the displaced Harmonic oscillator representation of the involved vibrational states [4]:

$$|i\rangle_g = \left(\frac{\beta}{\pi}\right)^{\frac{1}{4}} \left(2^i i!\right)^{-\frac{1}{2}} \exp\left(-\frac{1}{2}\beta\chi^2\right) H_i(\beta^{\frac{1}{2}}\chi) \tag{27}$$

$$|k\rangle_e = \left(\frac{\beta}{\pi}\right)^{\frac{1}{4}} \alpha^{\frac{1}{2}} \left(2^k k!\right)^{-\frac{1}{2}} \exp\left(-\frac{(\alpha\beta^{\frac{1}{2}}\chi + \delta)^2}{2}\right) H_k(\alpha\beta^{\frac{1}{2}}\chi + \delta)$$

where i and k are vibrational quantum numbers, χ is the dimensionless normal coordinate, δ is the difference between the equilibrium positions in the ground and excited electronic state, H_i is the i-th degree Hermite polynomial, and the α and β parameters are discussed below. With the help of Matlab (or other computer algebra systems) the Franck-Condon integrals required by Equation (26) can be thus evaluated numerically, so to produce the plot reported in Figure 3d) in the main text for the progression of the overtones of the α line of C_8. To determine the values of the Franck-Condon integrals the only required numerical value is $\delta_1 = 1.136$, which was determined as discussed above – see Table 4. The other parameters showing up in Equation (27) are $\alpha = \sqrt{\Omega_e/\Omega_g}$ and β. For the sake of simplicity, and to keep low the number of parameters, we neglect the change in curvature between the ground and excited state potential energy surface of the normal mode of the α-line. We therefore fix $\alpha = 1$. $\beta^{-1/2}$ is the returning point of the classical oscillator in its ground state [4]; it turns out that the Franck-Condon integrals are actually independent on the value of β – see for instance the analytical results reported in [4].

Figure 3d) in the main text also shows the comparison of the theoretical predictions of the progression of the intensities of the α-line of C_8 *vs.* the experimental counterparts that are tabulated in Table 5. Despite the simplifications of the theoretical model (harmonic approximation, neglect of curvature changes of the potential energy surface

Molecule	k		$\frac{I(0\to1)_{0k}}{I(0\to1)_{0k}}$	$\frac{I(0\to2)_{0k}}{I(0\to1)_{0k}}$	$\frac{I(0\to3)_{0k}}{I(0\to1)_{0k}}$	$\frac{I(0\to4)_{0k}}{I(0\to1)_{0k}}$	$\frac{I(0\to5)_{0k}}{I(0\to1)_{0k}}$
C_8	0	exp.	1.000	0.325	0.027	0.003	0.0001
		theo.	(1.000)	(0.261)	(0.045)	(0.006)	(0.0006)
	1	exp.	1.000	2.854	0.426	0.035	0.0254
		theo.	(1.000)	(3.846)	(2.021)	(0.529)	(0.0907)
	2	exp.	1.000	1.248	4.337	4.570	1.0228
		theo.	(1.000)	(0.009)	(0.397)	(0.421)	(0.1610)
C_{10}	0	exp.	1.000	0.291	0.034	0.007	–
		theo.	(1.000)	(0.275)	(0.050)	(0.007)	(0.0007)
	1	exp.	1.000	3.146	0.885	0.124	–
		theo.	(1.000)	(5.069)	(2.897)	(0.807)	(0.1460)
	2	exp.	1.000	1.231	0.931	0.820	–
		theo.	(1.000)	(0.030)	(0.343)	(0.423)	(0.1753)
C_{12}	0	exp.	–	–	–	–	–
		theo.	(1.000)	(0.287)	(0.054)	(0.007)	(0.0008)
	1	exp.	1.000	4.825	1.359	0.305	–
		theo.	(1.000)	(6.636)	(4.054)	(1.183)	(0.2224)
	2	exp.	1.000	0.078	0.262	0.134	–
		theo.	(1.000)	(0.055)	(0.298)	(0.416)	(0.1837)

Supplementary Table 5. **Experimental and calculated relative areas of the overtones of the α mode of polyynes at different resonances.** Relative areas of the overtones of the α-line of C_8, C_{10}, and C_{12} determined as peak area on the experimental resonance Raman spectra excited with UV light at the corresponding $|0\rangle_g \to |0\rangle_e$ (P, $k = 0$), $|0\rangle_g \to |1\rangle_e$ (I, $k = 1$), and $|0\rangle_g \to |2\rangle_e$ (II, $k = 2$).

of the excited state) and the simplified data analysis of the experimental data (peak height reading) the observed behavior of the relative Raman intensities is qualitatively well accounted for.

We have finally analyzed with the same approach as that used for C_8 the progression of the α line for the polyynes C_{10} and C_{12}. The results are reported in Table 5 and Figure 3e) and f) in the main text.

Supplementary References

1. Albrecht, A. C. On the Theory of Raman Intensities. *The Journal of Chemical Physics* **34,** 1476–1484. ISSN: 10897690 (1961).
2. Tabata, H., Fujii, M., Hayashi, S., Doi, T. & Wakabayashi, T. Raman and surface-enhanced Raman scattering of a series of size-separated polyynes. *Carbon* **44,** 3168–3176. ISSN: 00086223 (2006).

3. Tommasini, M. *et al.* Intramolecular vibrational force fields for linear carbon chains through an adaptative linear scaling scheme. *Journal of Physical Chemistry A* **111,** 11645–11651 (2007).

4. Ting, C.-H. Polarized Raman spectra-I. Selection rules. *Spectrochimica Acta Part A: Molecular Spectroscopy* **24,** 1177–1189. ISSN: 05848539 (1968).

5. Negri, F. *et al.* Resonance Raman contribution to the D band of carbon materials: Modeling defects with quantum chemistry. *The Journal of Chemical Physics* **120,** 11889–11900. eprint: https://doi.org/10.1063/1.1710853. https://doi.org/10.1063/1.1710853 (2004).

6. Wilson, E. B., Decius, J. C. & Cross, P. C. *Molecular Vibrations – The Theory of Infrared and Raman Vibrational Spectra* English (McGraw-Hill; New York, 1955).

7. Gierschner, J., Mack, H.-G., Lüer, L. & Oelkrug, D. Fluorescence and absorption spectra of oligophenylenevinylenes: Vibronic coupling, band shapes, and solvatochromism. *The Journal of Chemical Physics* **116,** 8596–8609. eprint: https://aip.scitation.org/doi/pdf/10.1063/1.1469612. https://aip.scitation.org/doi/abs/10.1063/1.1469612 (2002).

8. *Matlab's fsolve documentation* https://www.mathworks.com/help/optim/ug/fsolve.html. Accessed: 2021-08-13.
